# 

**Published:** 1836-07

**Authors:** 


### THE BRITISH AND FOREIGN MEDICAL REVIEW

OR

### QUARTERLY JOURNAL

OF

### PRACTICAL MEDICINE AND SURGERY

Lyceum libr[illegible]

EDITED BY

JOHN FORBES M.D. F.R.S.

AND

JOHN CONOLLY M.D.

EDITORS OF THE CYCLOPÆDIA OF PRACTICAL MEDICINE

ARMY MEDICAL LIBRARY
337714
MAR 25 '41
WASHINGTON, D.C., U.S.A.

VOL. II

APRIL—OCTOBER 1836

LONDON

SHERWOOD GILBERT AND PIPER

PATERNOSTER ROW

MDCCCXXXVI

W
1
BR 34

LONDON:

PRINTED BY J. AND C. ADLARD, BARTHOLOMEW CLOSE.

### BOOKS RECEIVED FOR REVIEW.

1. A Treatise on the Chemical, Medicinal, and Physiological Properties of Creosote, illustrated by Experiments on the lower Animals; with some Considerations on the Embalment of the Egyptians. Being the Harveian Prize Dissertation for 1836. By J. R. Cormac, Member of the Royal Medical and Physical Societies of Edinburgh.—Edinburgh, 1836. 8vo. pp. 154.

2. A Lecture on the Elements of Physical Education. By M. Louis Frechet. Read at the Philosophical Institutions at Bristol and Bath, 1835.—Bristol, 1836. 8vo. pp. 36.

3. Observations on Lord John Russell's Bill for registering Births, Deaths, and Marriages in England. By J. Yates, M.A. —London, 1836. 8vo. 2s.

4. On the Deaths of some eminent Philosophers of modern Time. By Sir Henry Halford, Bart.—London, 1836. 8vo. pp. 30.

5. The Obstetrician's Vade-Mecum; or, Aphorisms on Natural and Difficult Parturition, the Application and Use of Instruments, &c. By Thomas Denman, M.D. Considerably augmented, and arranged according to the present State of Obstetricy, by Michael Ryan, M.D. Ninth Edition.—London, 1836. Small 8vo. pp. 234; 17 plates.

6. Quackery; its Danger, Irrationality, and Injustice; the Causes of its Success; the best Means for its Suppression.—Bath, 1836. Pp. 18.

7. Guy's Hospital Reports. No. II. April, 1836. Edited by G. H. Barlow, M.A., and J. P. Babington, M.A.

8. Pathological Observations on the Diseases of the Placenta. Part I. Congestion and Inflammation. By J. Y. Simpson, M.D., President of the Royal Medical Society of Edinburgh. (Reprinted from the Edinburgh Medical and Surgical Journal, No.127.)—Edinburgh, 1836. 8vo. pp. 47.

9. Factory Statistics. The Official Tables appended to the Report of the Select Committee on the Ten-hour Factory Bill, vindicated in a Series of Letters. By the late M. T. Sadler, Esq. F.R.S.—London, 1836. 8vo. pp. 80.

10. Outlines of Human Pathology. By Herbert Mayo, F.R.S.—London, 1836. 8vo. pp. 330.

11. The Physiology of Digestion, considered with relation to the Principles of Dietetics. By Andrew Combe, M.D.—Edinburgh, 1836.—8vo. pp.332.

12. A new Dictionary of Medical Science and Literature. By R. Dunglison, M.D. &c. 2 vols.—Boston, 1833.

13. Medical Commentaries on Puerperal Fever, Vermination, and Water in the Head. By John Alexander, M.D.—London, 1836. 8vo. pp. 69.

14. A Discourse on self-limited Diseases. Delivered before the Massachusetts Medical Society, at their annual meeting, May 27, 1835. By Jacob Bigelow, M.D. —Boston, 1835. 8vo. pp. 48.

15. The Physiology of Respiration and Chemistry of the Blood, applied to Epidemic Cholera. By F. B. Joslin, M.D. (Extracted from the Transactions of the Medical Society of New York.—New York, 1835. 8vo. pp. 32.

16. An Address delivered to the Graduates in Medicine at the annual Commencement of the University of Maryland, in 1834. By Professor Dunglison.—Baltimore, 1834. 8vo. pp. 21.

17. Principles of Pathology and Practice of Physic. By John Mackintosh, M.D. Lecturer in Edinburgh, &c. With Notes and Additions, by S. G. Morton, M.D.—Philadelphia, 1836. 2 vols. 8vo.

18. Human Physiology; illustrated by Engravings. By Robley Dunglison, M.D. &c. Second Edition, with numerous additions and modifications.—Philadelphia, 1836. 2 vols. 8vo.

19. Researches in Medicine and Medical Jurisprudence. By J. B. Beck, M.D. &c. Second Edition.—New York, 1835. 8vo. pp. 258.

20. Manual of Practical Toxicology; condensed from Dr. Christison's Treatise on Poisons, with Notes and Additions. By J. F. Ducatel, M.D. &c.—Baltimore, 1833. 8vo.

21. On the Diagnosis of Diseases of the Chest; based upon the Comparison of their physical and general Signs. By W. W. Gerhard, M.D.—Philadelphia, 1836. 8vo. pp. 183.

22. Surgical Anatomy of the Arteries. By N. R. Smith, M.D., Professor of Surgery in the University of Maryland. 2d Edition, much enlarged; with twenty coloured Plates and many Woodcuts.—Baltimore, 1835. 4to. pp. 133.

[For the works No. 13—22 inclusive, we are indebted to the kindness of Professor Dunglison, of Baltimore.]

23. Isis Revelata: an Inquiry into the Origin, Progress, and present State of Animal Magnetism. By J. C. Colquhoun, Esq., Advocate, F.R.S.E.—Edinburgh, 1836. 2 vols.

24. Cyclopædia of Anatomy and Physiology. Part VI. (Cavity-Cilia.) May, 1836.

25. Lectures on the Nervous System, and its Diseases. By Marshall Hall, M.D. F.R.S. L. & E.—London, 1836. 8vo. pp. 171.

26. The Clinique Médicale; or, Reports of Cases. By G. Andral. Translated by D. Spillan, M.D. Part IV. Diseases of the Abdomen.—London, 1836. 5s.

27. Eupædia; or, Letters to a Mother on the watchful Care of her Infant; in reference to Diet, Clothing, Air, Exercise, Medicine, &c. By a Physician.—London, 1836. 12mo. pp. 144.

28. Essay on the Disorders incident to Literary Men, and on the best Means of preserving their Health. Read before the Royal Society of Literature. By W. Newnham, Esq. M.R.S.L.—London, 1836. 8vo. pp. 36.

29. Illustrations of the Botany and other Branches of the Natural History of the Himalayan Mountains, and of the Flora of Cashmere. By J. F. Royle, Esq. F.L.S. &c. Part IX.

30. On Disease of the Hip-Joint, with some Observations on Deformities of the Chest. By William Coulson, M.R.C.S. &c. —London, 1836. 8vo. pp. 32; with a Plate.

31. An authentic Report of an introductory Lecture on the important Subject of Medical Reform; delivered in the School of Anatomy, Medicine, and Surgery, Peter street, Oct. 30, 1835. By Andrew Ellis, Esq.—Dublin, 1836.—8vo. pp. 63.

32. Observations on the Medical and Surgical Agency of the Air-Pump. Read before the British Association in 1835. By Sir James Murray, M.D.—Dublin, 1836. 8vo. pp. 63.

33. Lectures on Subjects connected with Clinical Medicine. By P. M. Latham, M.D.—London, 1836. 8vo. pp. 322.

34. An Enquiry into the Pathology, Causes, and Treatment of Puerperal Fever: being an Essay for which the Fothergillian Gold Medal was conferred on the Author by the Medical Society of London, in March, 1835. By G. Moore, F.R.C.S. and F.R.M.C.S.—London, 1836. 8vo. pp. 247.

35. Address of Earl Stanhope, President of the Medico-Botanical Society, for the Anniversary Meeting, Jan. 1836.—London. 8vo. pp. 30.

36. History and present State of Brislington House near Bristol, an Asylum for the Cure and Reception of Insane Persons. Conducted by F. and C. Fox, M.DD.—Bristol, 1836. 4to. pp. 10; with Plates.

37. Report on the Medical Institutions of Ireland. By W. P. Borrett, M.D.—London, 1836. Pp. 30.

38. The Transactions of the Provincial Medical and Surgical Association. Vol. IV.—London, 1836. 8vo. pp. 578.

39. On the Antidotal Treatment of the Epidemic Cholera. By John Parkin.—London, 1836. 8vo. pp. 112.

40. A Popular View of Homœopathy. By the Rev. T. R. Everest. Second Edition.—London, 1836. Pp. 151.

41. Cataract: a familiar Description of its Nature, &c. By John Stephenson, Esq. Second Edition.—London, 1836. 12mo. pp. 136.

FOREIGN.

1. Diagnostisch-praktische Abhandlungen aus dem Gebiete der Medicin und Chirurgie, durch Krankheitsfälle erlautert vom Dr. Löwenhardt. Erster Theil.—Prenslau, 1835. 8vo. pp. 352.

2. Ueber Volkskrankheiten und deren bekämpfung. Von Ernst L. H. Lebenheim, Dr. Med. et Chir. &c.—Hamburg, 1836. 8vo. pp. 144.

3. Die Influenza. Ein historischer und aetiologischer Versuch von Henreich Schweich, Doctor der Medicin und Chirurgie. Mit einer Vorrede von Dr. J. F. C. Hecker, M.D. &c.—Berlin, 1836. 8vo. pp. 188.

4. Zur Diagnostik der Lungen-und Herzkrankheiten mittelst physicalischer Zeichen: mit besonderer berücksichtigung der Auscultation und Percussion. Von Dr. P. J. Philipp.—Berlin, 1836. 8vo. pp. 358.

5. S. A. Steinii Tabulæ Anatomicæ præcipuarum humani corporis regionum, in quibus graviores operationes chirurgicæ suscipiuntur. Fasciculi primi Pars prior, Pars posterior.—Hauniæ. Fol. 1831-33.

6. Thymi in homine ac per seriem animalium descriptio anatomica, pathologica, et physiologica, iconibus xxxiv illustrata. Auctore F. C. Haugsted, M.D.—Hafniæ, 1832. 8vo. pp. 286.

7. Beiträge zur Philosophie der Seele. Von C. F. Flemming, M.D.—Berlin, 1830. 2 vols. 8vo. (From Dr. Oppenheim.)

8. Curbilder, mit Bezug auf Cholera. Vom Dr. Krüger-Hansen.—Rostock, 1831. 8vo. (From Dr. Oppenheim.)

9. Erfahrungen über Homöopathie unter den augen homoopathischer Aertze gesammelt. Von Dr. C. Friedheim.—Berlin, 1835. Pp. 80.

10. Beiträge zur geschichte des Petechial-typhus. Von Karl Pfeufer, M.D.—Bamberg, 1831. 8vo.

11. Dissertatio de Signis, mortem hominis absolutam ante putredinis accessum indicantibus.—Hauniæ, 1833. 8vo. (From Dr. Otto.)

12. Wider die Mystification in der Medicin. Von Dr. C. N. E. Bischoff.—Bonn, 1830. 8vo.

13. Anviisning til at Kjende Lunge- og Hierte-Sygdomme ved Percussion og middelbar Auscultation. Ved Dr. Med. S. Trier.—Kjöbenhavn, 1830. 8vo. (From Dr. Otto.)

14. J. A. Paris, M.D. Diætetik. Oversat af Engelsk, af F. V. Mansa, M.D. (Translation of Dr. Paris's work on Diet, into Danish.)—Kiöbenhavn, 1832. 8vo. (From Dr. Otto.)

15. Relatorio dos Trabalhos da Sociedade de Medicina do Rio de Janeiro & pelo Dr. L. V. De-Simoni.—Rio de Janeiro, 1831-2. 8vo.

16. Ueber das Verhältniss der Nervösen Fieber zu Cholera und Intermittens. Von Joseph Heine, M.D.—Munchen, 1833. (From Dr. Oppenheim.)

17. Education Physique des jeunes Filles, ou Hygiène de la Femme avant le Mariage. Par A. M. Bureaud-Rioffrey, M.D. &c.—Paris, 1835. 8vo. pp. 352.

18. Die Erkenntniss und Heilung der Ohrenkrankheiten. Von Dr. Wilhelm Kramer.—Berlin, 1836. 8vo. pp. 400.

19. Discorso sobre as Molestias que mais affligem a classe pobre do Rio Janeiro. Pelo J. M. Da Cruz Jobim, Medico do Hospital da Misericordia, &c.—Rio de Janeiro, 1835. 8vo. p. 35.

20. Compte Rendu des Travaux de l'Ecole de Médecine d'Abou-Zabel, (Egypte,) &c. Par Clot-Bey, M.D. &c.—Paris, 1833.

21. Epitome Therapiæ Generalis; in usum discipulorum scripsit G. C. B. Suringar, M.D. &c.—Amstelodami, 1834. 8vo. pp. 150.

22. Epidemia Vaiuloso del 1829, in Torino, &c. Per T. D. Griva.—Torino, 1831. 8vo. pp. 249.

23. Die Homöopathie und Allopathie auf der wage von Krüger-Hansen.—Güstrow, 1833. 8vo. (From Dr. Oppenheim.)

24. Zweiter Anatomischer Bericht der Pathologischen Präparate, &c. Von Dr. C. T. Tortual.—Münster, 1833. 4to.

25. Ueber die Augenkrankheiten welche in der Belgische Armee herrscht. Von J. C. Jüngken, M.D.—Berlin, 1834. 4to.

26. Heelkundige Mengelingen, door J. F. Kerst, Chirurgiæ Doctor, &c.—Utrecht, 1835. 8vo. pp. 256.

27. Schets der Heelkunde tot leiddraad voor zijne Lessen uitgegeven door C. B. Tilanus, Med. et Chir. Doct. &c.—8vo. pp. 193. Amsterdam, 1835.

28. Ueber Paralyse der Inspirations-Muskeln. Von Dr. Louis Stromeyer, Königl. Hofchirurgus, &c.—Hannover, 1836. 8vo. pp. 144.

